# *Culex* species diversity, susceptibility to insecticides and role as potential vector of Lymphatic filariasis in the city of Yaoundé, Cameroon

**DOI:** 10.1371/journal.pntd.0007229

**Published:** 2019-04-03

**Authors:** Elysee Nchoutpouen, Abdou Talipouo, Borel Djiappi-Tchamen, Landre Djamouko-Djonkam, Edmond Kopya, Carmene Sandra Ngadjeu, Patricia Doumbe-Belisse, Parfait Awono-Ambene, Sevilor Kekeunou, Charles Sinclair Wondji, Christophe Antonio-Nkondjio

**Affiliations:** 1 Laboratoire de Recherche sur le Paludisme, Organisation de Coordination pour la lutte Contreles Endémies en Afrique Centrale (OCEAC), Yaoundé, Cameroon; 2 Faculty of Science, University of Yaoundé I, Yaoundé, Cameroon; 3 Vector Borne Infectious Disease Unit of the Laboratory of Applied Biology and Ecology (VBID-LABEA), Department of Animal Biology, Faculty of Science, University of Dschang, Dschang, Cameroon; 4 Vector Biology Liverpool School of Tropical medicine Pembroke Place, Liverpool, United Kingdom; Federal University of Agriculture, NIGERIA

## Abstract

**Background:**

*Culex* species are widespread across Cameroon and responsible for high burden of nuisance in most urban settings. However, despite their high nuisance, they remain less studied compared to anophelines. The present study aimed to assess *Culex* species distribution, susceptibility to insecticide, bionomics and role in Lymphatic Filariasis (LF) transmission in the city of Yaoundé.

**Methods:**

Mosquito collections were conducted from March to December 2017 using Centre for Disease Control light traps (CDC-LT), human landing catches (HLC) and larval collections. Mosquitoes were identified using morphological identification keys. Mosquitoes from the *Culex pipiens* complex were further identified using Polymerase Chain Reaction (PCR) to assess the presence of sibling species. Bioassays were conducted with 2–5 day-old unfed females to assess mosquito susceptibility to DDT, permethrin, deltamethrin and bendiocarb following WHO guidelines. Dead, control and surviving mosquitoes from bioassays were screened by PCR to detect the presence of knockdown resistance (*kdr*) alleles. Pools of mosquitoes were examined by PCR to detect the presence of *Wuchereria bancrofti*.

**Results:**

A total of 197,956 mosquitoes belonging to thirteen species were collected. The density of mosquito collected varied according to the collection methods, districts and seasons. *Culex quinquefasciatus* emerged as the most abundant and the only species of the *Culex pipiens* complex in Yaoundé. *Culex* species were found breeding in different types of breeding sites including polluted and unpolluted sites. All *Culex* species including *Cx antennatus*, *Cx duttoni*, *Cx perfuscus* and *Cx tigripes* were found to be highly resistant to permethrin, deltamethrin and DDT. *Culex quinquefasciatus* was also found to be resistant to bendiocarb. A high frequency of the West Africa *kdr* allele was recorded in resistant *Cx*. *quinquefasciatus*. Out of the 247 pooled samples of 25 *Culex* spp. examined for the presence of *Wuchereria bancrofti*, none was found infected.

**Conclusion:**

The study confirms the high adaptation of Culex species particularly *Culex quinquefasciatus* to the urban environment and no implication of this species in the transmission of LF in Yaoundé Cameroon. Culex species predominance in urban settings highlight potential transmission risk of West Nile and rift valley fever in Yaoundé.

## Author summary

*Culex* species are highly prevalent in both urban and rural settings in Cameroon and are responsible for high nuisance and transmission of pathogens such as Wuchereria *bancrofti* and arbovirus. Despite the important epidemiological role, that Culex could play, they are still less studied. The current study was conducted to assess Culex species distribution, susceptibility to insecticide and role in *W*. *bancrofti* transmission in the city of Yaoundé. Mosquito collection was conducted using three collection methods human landing catches, CDC light traps and larval collection. Once collected, mosquitoes were identified using morphological identification keys and PCR diagnostic tools. They were later processed to determine their infection status. Bioassays with *Culex* females of 2 to 5 days old were conducted to determine their susceptibility level to different insecticide families. *Culex quinquefasciatus* emerged as the most abundant species. Up to 13 different culicine species were recorded. *Culex* species were recorded to be highly resistant to DDT, permethrin and deltamethrin. A high frequency of the West Africa *kdr* allele was recorded. No mosquito was detected to be infected by LF.

The study confirms the need for further xenomonitoring activities in order to control the risk of outbreaks due to Culex mosquitoes in the city of Yaoundé.

## Introduction

*Culex* species are the most widespread mosquito species across the world [1]. They are known to be highly opportunistic feeding on both humans and animals, a behaviour which increases their potential to transmit zoonotic diseases and makes them important threat to public health [2]. *Culex* have over decades adapted to human made habitats [3]. One of the most important group in the *Culex* genus is *Culex pipiens* complex which comprises six members: *Cx*. *quinquefasciatus* Say, *Cx*. *pallens* Coquillet, *Cx*. *australicus* Dobrotworsky & Drummond, *Cx*. *globocoxitus* Dobrotworsky, *Cx*. *pipiens* Linneaus and *Cx*. *molestus* Forskll [4, 5]. Species of the *Cx*. *pipiens* complex particularly *Cx*. *quinquefasciatus* are widespread and predominant in the urban environment notably in Africa where suitable environmental conditions created by rapid unplanned urbanization is contributing to their proliferation [6–9]. *Culex quinquefasciatus* can be found in all types of water collections including temporary or permanent stagnant water bodies such as drains, septic tanks, wet pit latrines, organically polluted sites, puddles [10] and has emerged as the most common mosquito species in major African cities [11–13]. In addition to nuisance that *Culex* species could induce, they also transmit diseases such as Japanese and Saint Louis encephalitis, Rift valley fever, West Nile Virus and lymphatic filariasis (LF) [14, 15]. The later caused by the parasite *Wuchereria bancrofti* is largely prevalent in Asia and sub-Saharan Africa and is consider as one of the leading causes of long term disability in the World [16–18]. In Cameroon, Lymphatic filariasis is considered to be endemic with mean prevalence level (ICT>1%) estimated at 3.3% countrywide [19]. LF is among the neglected tropical diseases targeted for elimination by the World Health Organization by 2020 using mass drug administration (MDA) [20]. Although direct implication of *Culex* species in the transmission of LF in West and Central Africa is still not well documented [21, 22], in East Africa, *Culex* species particularly *Cx*. *quinquefasciatus* is known to have a major role in LF transmission [23, 24]. With changing climate associated to increased traffic between East and West African countries and rapid expansion of this species in urban settings, it is becoming crucial to assess the role of *Culex* species in the transmission of diverse diseases. In most cities in Cameroon *Cu*lex are the main species causing the highest nuisance in the population. Household survey conducted in the cities of Douala and Yaoundé indicated that in addition to treated nets, tools such as insecticide spray, coils, screen are permanently used by urban dwellers to fight against mosquito nuisance [25, 26]. In the cities of Douala and Yaoundé, high pyrethoid resistance in *An*. *gambiae* populations was reported [27–29], whereas for *Culex* species there is still not enough data on their bionomic in the urban environment. Data on species composition, spatial distribution, susceptibility to insecticides and implication in diseases transmission are all lacking. This information is of paramount importance in the perspective of integrated vector management and insecticide resistance management [30]. Also, understanding the bionomic and distribution of *Culex* species could enable understanding the epidemiology of diseases that they transmit and to establish sustainable surveillance and control programmes. The present study assesses the distribution, susceptibility status to insecticides and epidemiological role of *Culex* species before the implementation of a larval control trial in the city of Yaoundé.

### Methods

**Study site**- The study was conducted in Yaoundé (03°52’N; 11°31’E), the capital city of Cameroon from March to December 2017. The city has a population estimated at 2.8 million inhabitants. Yaoundé belongs to Guinean subequatorial climate type, characterized by four distinct seasons: the short rainy season (Mars-June), the short dry season (June-July), the long rainy season (August-November) and the long dry season (November-February). The city receives annually over 1600 mm of rainfall and the annual average temperature is 24°C. Yaoundé is located about 750 m above sea level and surrounded by many hills. Although occurring at very low endemicity, human infection by Wuchereria *bancrofti* was estimated at 2.3% during surveys conducted between 2009–2010 in Yaoundé and it surroundings [19].

The study was conducted under the ethical clearance N° 2016/11/832/CE/CNERSH/SP delivered by the Cameroon National Ethics Committee for Research on Human Health (CNERSH) Ref N°D30-172/L/MINSANTE/SG/DROS/TMC of 4 April 2017. For human landing catches all adult men who took part in the collection signed a written informed consent form before being enrolled in the study as recommended by the validated protocol and were given free malaria prophylaxis.

**Mosquito’s collection and breeding sites characterization**- Adult and immature stages of Culicine mosquitoes were sampled in 32 districts of Yaoundé. Culicine collections were undertaken in the context of a big survey intended to assess mosquito distribution and malaria transmission pattern in the city of Yaoundé before a larval control trial and will allow in the future additional analysis with more data. Adult mosquitoes were collected using CDC light traps (CDC-LTs) and Human Landing Catches (HLCs) from 7pm to 6am. All potential larval breeding sites were inspected and positive sites (with at least one Culicine larvae or pupae) recorded. Three dips were undertaken for small breeding sites of less than 1 m^2^; and 5 to 10 dips were undertaken in breeding sites of more than 1m^2^. The average larval density (N) was estimated. Once collected larvae were classified according to their stages: early instars larvae (L1&L2) and late instars (L3&L4 and pupa). Other parameters measured included the type of breeding sites sampled (stagnant water pools, gutters, well, tyre print, footprint, pit latrine….), depth, the status organically polluted or not, the distance to the nearest house, the presence/absence of predators, the proportion of water surface covered by vegetation or algae.

Larvae collected were kept in plastic containers and brought to the insectary for rearing. After emergence, adult mosquitoes were identified to species level under a binocular magnifying glass using morphological identification keys [31–33]. For mosquitoes collected using either CDC-LTs or HLC, a subsample of 50 culicine specimens per district was randomly selected for identification during each collection month. All mosquitoes collected were stored at -20°C for further molecular analyses.

**Susceptibility tests to insecticides**-Bioassays were performed with 2–5 days old females emerging from larval collection. Mosquitoes were tested against permethrin 0.75%, DDT 4%, bendiocarb 1% and deltamethrin 0.05% following WHO guidelines [34].

For each test, batches of 25 mosquitoes per tube were exposed to impregnated papers for 1 hour. The number of mosquitoes knocked down by the insecticide was recorded every 10 minutes during exposure. After exposure, mosquitoes were fed with a 10% glucose solution and the number of dead mosquitoes was recorded 24 hours post-exposure. Mosquitoes used as controls were exposed to untreated papers. The mortality rates were corrected using the Abbot formula [35] whenever the mortality rate of the controls was between 5 and 20%. Susceptibility and resistance levels were assessed according to WHO criteria [34]. At the end of the assay, mosquitoes were classified into three different groups: 98%-100% mortality indicates susceptibility, 80%-97% mortality suggests possible resistance that needs to be confirmed, <80% mortality suggests resistance.

#### Molecular identification of members of *Culex* pipiens complex

To identify members of *Culex pipiens* complex, DNA was extracted from whole adult female mosquitoes according to the methods described by Livak [36], DNA extracted was used to run a multiplex PCR assay [37]. The locus CQ11 was used to distinguish between *Cx*. *pipiens*, *Cx*. *quinquefasciatus* and *Cx*. *pallens*. PCR amplification reactions were carried out in 15μl volume reaction mix, containing 10xPCR buffer, 250μM of each DNTP, 1.7mM MgCl2, 0.15mM of bovine serum albumin, one unit Taq polymerase (Applied Biosystems), 2μl of genomic DNA, 11.6nM of each of following primers: ACE pip for *Cx*. *pipiens*, ACE quin for *Cx*. *quinquefasciatus*, ACE pall for *Cx*. *pallens* and B1246s (Table 1). The PCR conditions were 5 min at 94°C followed by 30 sec at 94°C, 30 sec at 62°C and 1 min at 72°C for 35 cycles and 5 min at 72°C for the final extension. The PCR products were then separated by electrophoresis on 1.5% agarose gel with Midori green and visualized under ultraviolet light.

**Table 1:** Primers for molecular identification of members of the *Culex pipiens* complex.

Primers	5’-3’ sequences	Product size with B1246s (bp)
ACEpip	5’-GGA AAC AAC GAC GTA TGT ACT-3’	610
ACEpall	5’-ATG GTG GAG ACG CAT GAC G-3’	478
ACEquin	5’-CCT TCT TGA ATG GCT GTG GCA-3’	274
B1246S	5’-TGG AGC CTC CTC TTC ACG G-3’	

ACE pip primer for *Cx*. *pipiens*, ACE quin primer for *Cx*. *quinquefasciatus*, ACE pall primer for *Cx*. *pallens* and B1246S (reverse primer).

#### Detection of kdr mutation

The PCRs were performed to detect the knock-down resistance (*kdr*) mutation in a single mosquito following the protocol of Martinez-Torrez et *al*. [38] with minor modifications concerning PCR conditions: 5 min at 94°C, 30 sec at 94°C, 35 sec at 57°C, 45 sec at 72°C and 5 min at 72°C. Two separated PCRs reactions for each mosquito were run in parallel, one to detect resistant alleles (the leucine-phenylalanine substitution) and the other to detect susceptible alleles (the wild allele). During the first reaction, the primers Cdg1, Cdg2 and Cdg3 were combined and in the second one, Cdg3 was replaced by Cdg4. The PCR conditions were 5 min at 94°c for the first cycle followed by 30 sec at 94°c, 35 sec at 57°c and 45 sec at 72° C for 35 cycles and 5 min at 72°C for the final extension. The PCR products were then separated by electrophoresis on 1.5% agarose gel with Midori green and visualized under ultraviolet light.

#### Detection of *Wuchereria bancrofti*

Pools of 25 Culex mosquitoes each were examined for the presence of *W*. *bancrofti* using PCR [39]. DNA was extracted from the pooled mosquitoes according to the methods described by Livak [36]. PCR was conducted using two specific primers of *W*. *bancrofti*, 24.3 nM NV1 and 33.0 nM NV2, targeting a highly repeated DNA sequences (SsPI repeat) of *W*. *bancrofti*. PCR analysis were conducted in a final reaction mixture volume of 15 μl containing 0.12μl of Hot-Start Tempase polymerase, 0.51μl of each two primers and 2μl of DNA extract. The PCR conditions were as follows 10min at 94°C followed by 35 cycles of denaturation during 30sec at 94°C, annealing at 54°C for 45 sec, extension at 72°C for 45 seconds and final extension at 72°C for 10 minutes. The amplified PCR products were then separated by electrophoresis on 1.5% agarose gel with Midori green and visualized under ultraviolet light.

#### Statistical analysis

The following entomological indicators were calculated: trap visiting rate (TVR) representing the number of *Culex* visiting a trap per night; Human landing rate (HLR) representing the number of *Culex* that land on a man per night; *Culex* emergence rate (CER) representing the number of *Culex* emerged per 100 larvae reared to the adult stage. Direct analyses were performed using the SPSS V 20.0 software to estimate means, proportions and for comparisons. Pearson’s Chi square analysis was performed to compare the mortality rate between various *Culicinae* species. The P-value below 0.05 was considered statistically significant. To assess the level of correlation between breeding habitats, physical characteristics and densities of *Culicinae* larvae in the breeding sites, a multiple component analysis was applied using *FactoMineR* package of the software R version 3.4.0 [40].

### Results

#### Mosquito composition and abundance

A total of 197,956 mosquitoes were collected using CDC-LTs, HLC and larval collections then classified according to genus and species (Table 2). Mosquitoes from larval collections were reared up to the adult stage before being identified. Mosquitoes collected included, *An*. *gambiae sl*., *An*. *funestus*, *Culex spp*., *Aedes spp*., *Mansonia spp*., and *Coquilletidia spp*. *Culex* species were by far the most abundant group. The majority of mosquitoes (n = 169,590) were collected using 1,917 trap nights. The total number of mosquitoes collected with Human landing collections (25,115) was obtained using 320 human night collectors. Of the 1,234 *An*. *gambiae* sl processed by PCR, 92% were *An*. *coluzzii* and 8% were *An*. *gambiae*.

**Table 2:** Composition of the mosquito fauna collected in Yaoundé from March to December 2017.

Species	HLC (%)	CDC (%)	Larval (%)	Overall (%)
*An*. *gambiae* s.l.	2,368 (9.4)	3,789 (2.2)	78 (2.4)	6,235 (3.1)
*An*. *funestus*	79 (0.3)	498 (0.3)		577 (0.3)
*Culex* spp.	22,531 (89.7)	164,151 (96.8)	3,088 (95)	189,770 (95.9)
*Aedes* spp.	29 (0.1)	232 (0.1)	85 (2.6)	346 (0.2)
*Mansonia* spp.	106 (0.4)	920 (0.5)		1,028 (0.5)
*Coquilletidia* spp.	2			
Total	25,115 (100)	169,590 (100)	3,251 (100)	197,956 (100)

#### Culicine species diversity

Of the 191,144 culicine species collected, 13,982 were identified down to species level using morphological identification keys. A total of 221 *Culex* species morphologically identified as belonging to the *Cx*. *pipiens* complex were further processed by PCR and confirmed as *Cx*. *quinquefasciatus* after molecular identification.

For species identification (of mosquitoes collected with HLC and CDC LT), a sample of at least 50 mosquitoes per district were randomly selected during each collection period. Thirteen culicine species were recorded. Twelve of the species were recorded using CDC light traps (Table 3). *Culex quinquefasciatus*, *Cx*. *perfuscus*, *Cx*. *duttoni*, *Cx*. *antennatus* were the most commonly recorded in the *Culex* genus while, *Ae*. *aegypti*, *Ae*. *albopictus* and *Ae*. *furcifer* were the main *Aedes* species found. *Mansonia Africana* and *Man*. *uniformis* were also recorded (Table 3).

**Table 3:** Density estimates of culicine species recorded using different sampling techniques.

Mosquito species	CDC light trap	HLC	Larval collection
*Cx quinquefasciatus*	++++	+++	++++
*Cx duttoni*	++	+	+++
*Cx perfuscus*	+++	+	++
*Cx antennatus*	+	+	+++
*Cx tigripes*	+	-	+
*Cx poicilipes*	+	-	-
*Cx univittatus*	-	-	+
*Ae*. *albopictus*	++	-	-
*Ae*. *Aegypti*	+	-	-
*Ae*. (Diceromyia) furcifer	+	-	+
*Man*. *uniformis*	++	+	-
*Man*. *Africana*	+	-	-
*Coquilletidia sp*.	+	-	-

Cx: Culex; Ae: Aedes, Man: Mansonia

+ (1≤N<100); ++ (100≤N<200); +++ (200≤N<300); ++++(N>300)

#### Density of culicine species collected according to sampling methods

Of the 13, 982 mosquitoes identified, 9,613 were derived from CDC- LTs collections, 1,236 from HLC and 3,133 from larval collections. Concerning larval collections, identifications were conducted on specimens emerging from at least ten sites per district. The densities of almost all species collected greatly varied according to the collection method except for *Cx*. *quinquefasciatus*. *Culex antennatus*, *Cx*. *univittatus* and *Cx*. *tigripes* were more abundant in larval collections whereas *Ae*. *albopictus*, *Ae*. *aegypti*, *Man*. *uniformis* and *Man*. *africanus* were mainly collected using CDC- LTs and/or HLC (Table 3). The overall average trap visiting rate for Culex species was 22.12 mosquitoes/trap/night (mos/trap/night) indoor and 10.16 mos/trap/night outdoor. The average human landing rate for *Culex spp* was 75.1 mosquitoes/man/night indoor and 79.58 mos/man/night outdoor. The mean survival rate from larvae to adult for Culex species was between 74% and 90% when the evaluation started with third instar larvae.

#### Spatial distribution of various mosquito species

The distribution of culicine species in different districts across the city of Yaoundé was also assessed. *Culex quinquefasciatus*, *Cx*. *perfuscus* and *Cx*. *duttoni* were the most abundant representing 79.4%, 7.3% and 7.9% respectively of the total mosquitoes identified. In the majority of districts a minimum of 6 different *Culex* species were recorded (see S1 Data).

#### Seasonal distribution of culicine species

**Fig 1:**
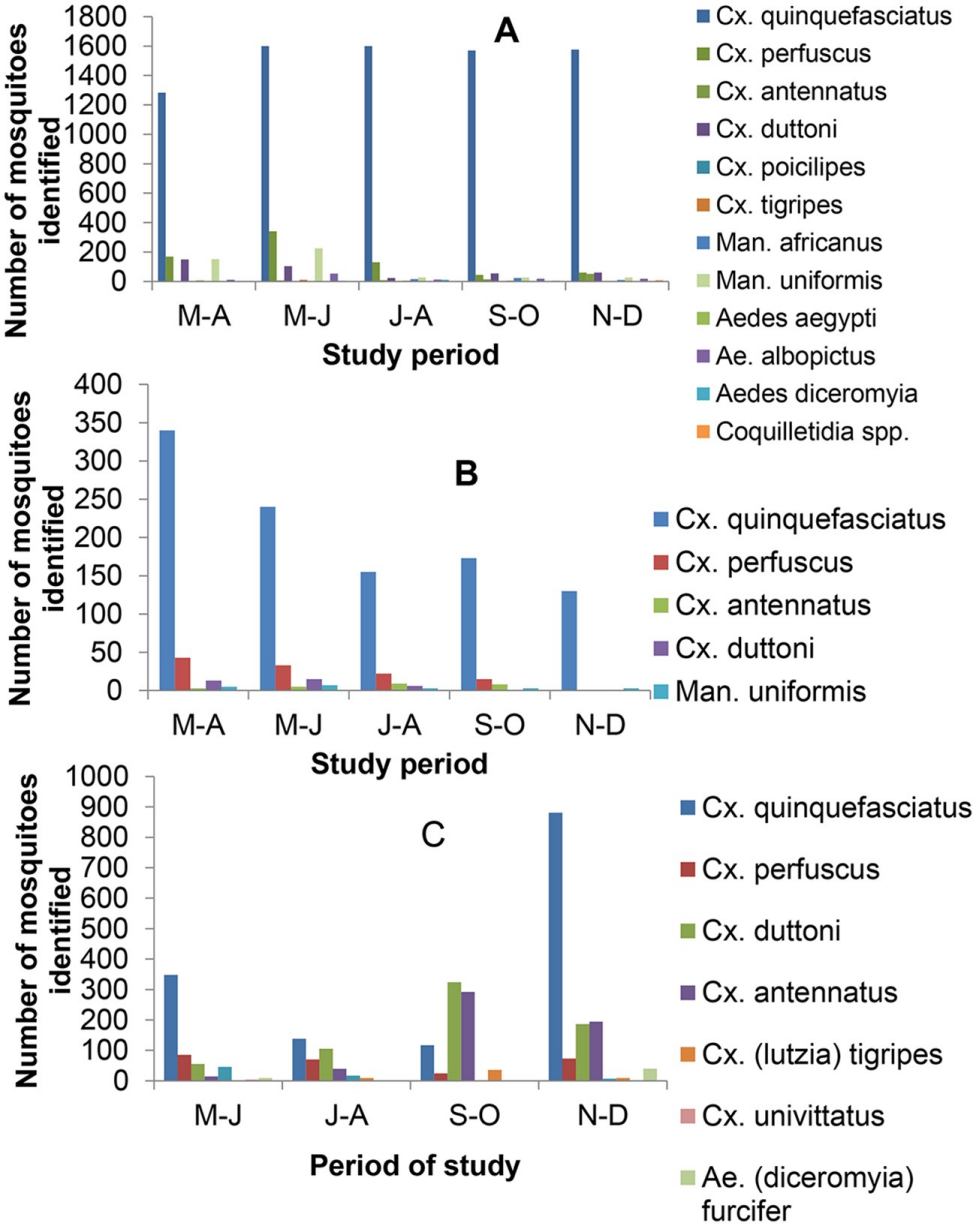
Seasonal distribution of mosquitoes collected using CDC Light trap (A), HLC (B) and larval collection (C) in districts of the city of Yaoundé. M-A, March-April; M-J, May-June; J-A, July-August; S-O, September-October; N-D, November-December.

Mosquito collections were undertaken at different months to assess seasonal variation in species composition (Fig 1). When CDC-LTs collections or HLC were considered, no important variation was detected between months. *Culex quinquefasciatus* was always the predominant species all year round. When larval collections were considered, significant variations were recorded between months. *Culex quinquefasciatus* was the dominant species during the months of May and June and November and December while it was replaced by *Cx*. *duttoni* in September and October.

#### Physical and biological factors affecting *Culex* mosquito distribution in breeding sites

**Fig 2:**
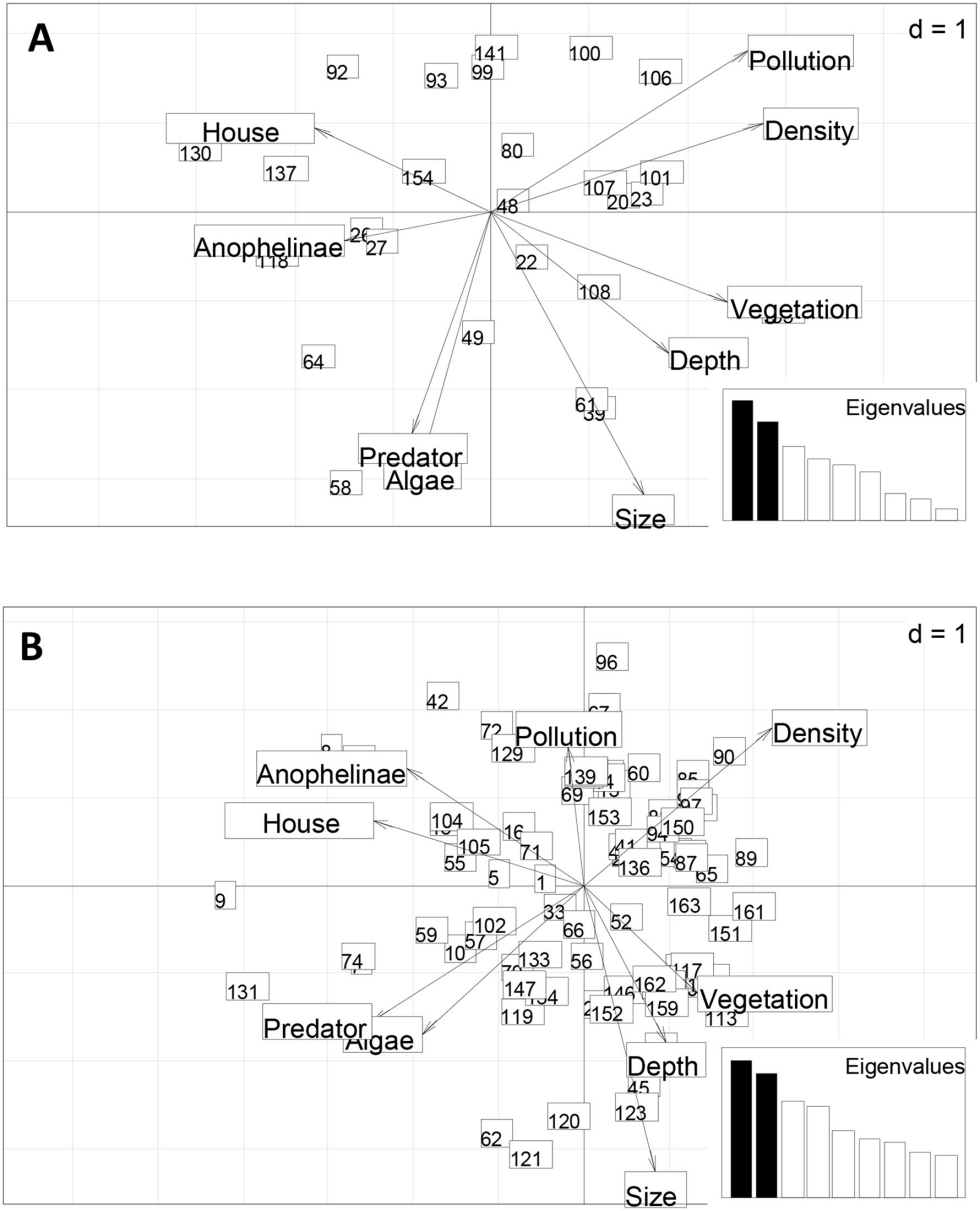
Multiple component analysis showing relationship between early instar culex larvae (A), late instar culex larvae (B) densities and some physical characteristics of their breeding sites in Yaounde. Legend1,2, 3, 4….: refer to breeding sites, Size = size of the breeding site, Predator = presence of predators, Density = the number of larvae in each breeding site, Pollution = presence of organic pollution; Anophelinae: presence of anophelinae larvae, Algae = presence of algae, House = distance of breeding sites to houses, Depth = the depth of the breeding site.

*Culex* larvae were found in various types of breeding sites including stagnant water pools, gutters, wells, tyre prints, footprints, pit latrine etc. In most of the breeding sites *Culex* larvae were found in sympatry with Anopheline larvae. A multicomponent analysis was conducted to assess association between *Culex* larval stages densities and breeding sites characteristics (size and depth of the breeding sites, organic pollution, presence/absence of predators, presence/absence of vegetation, presence/absence of Anopheline larvae and presence of houses). No significant association was recorded between culicine larva densities and any of the recorded physical characteristics of the breeding site. Early instar larvae (L1 & L2) presence was found to be negatively correlated to factors such as the presence of predators and algae and positively correlated to pollution (Fig 2). For late instar larvae (L3, L4), no factor appeared to be significantly associated with their distribution (Fig 2). The association of *Culex* & Anopheline mosquitoes was less common for early instars but was more frequent for late instars.

#### Susceptibility to insecticides

**Fig 3:**
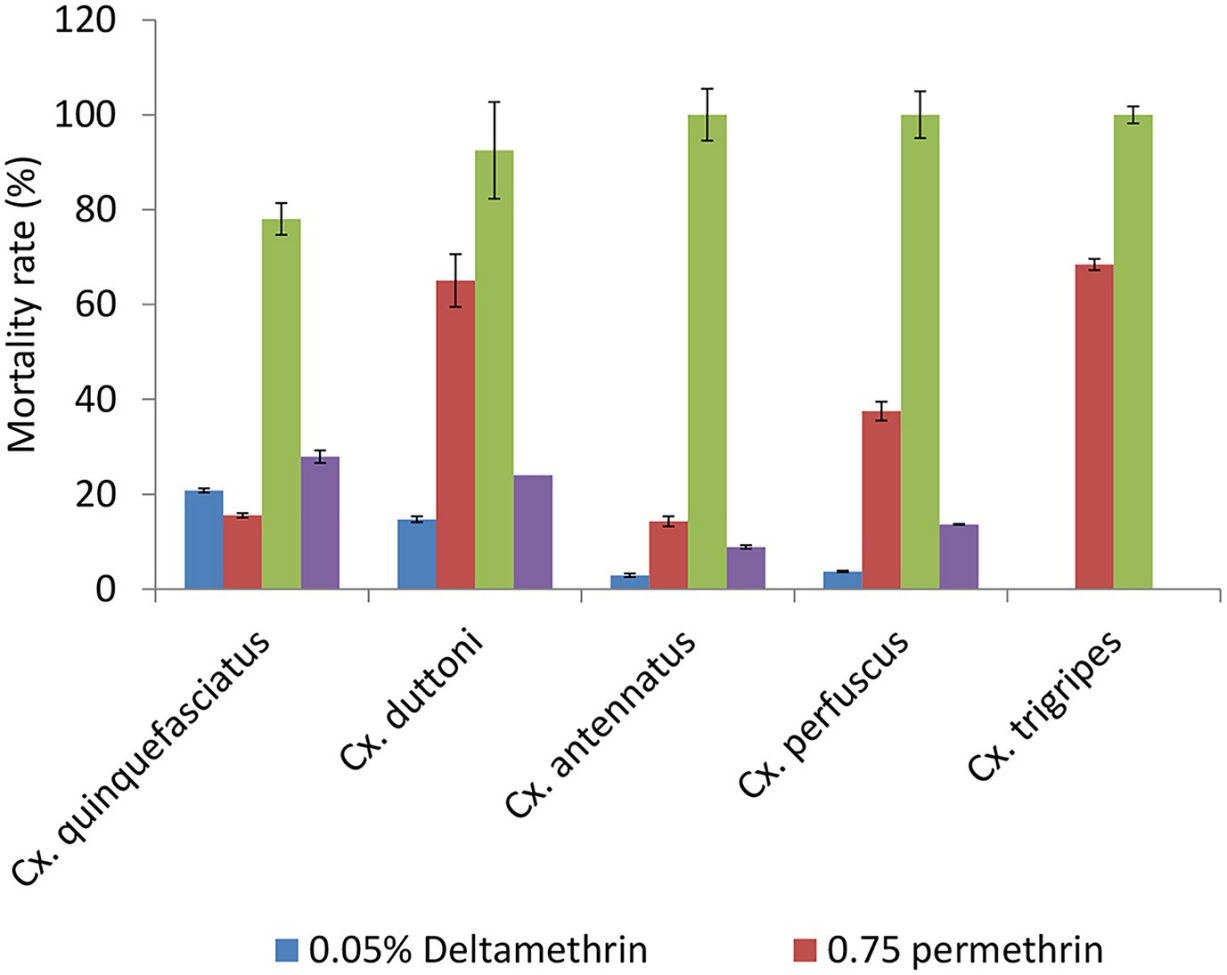
Mortality rate of culex species exposed to different insecticides. Error bars represent standard error of the mean.

A total of 3,545 females were exposed to either 4% DDT, 0.75% permethrin, 1% bendiocarb or 0.05% deltamethrin. *Culex* species tested included *Cx*. *quinquefasciatus*, *Cx*. *antennatus*, *Cx*. *duttoni*, *Cx*.*perfuscus* and *Cx*. *tigripes*. All *Culex* species tested were found to be highly resistant to permethrin (mortality rate ranging from 14.25% to 66.05%), deltamethrin (mortality rate ranging from 2.91% to 20.78%) and DDT (mortality rate ranging from 8.87% to 27.91%). *Culex quinquefasciatus* was also found to be resistant to bendiocarb (Fig 3).

#### Kdr mutation in *Culex quinquefasciatus*

A total of 201 mosquitoes including controls and survivors were screened by PCR to detect the presence of the *kdr* alleles; 122 were found carrying the kdr allele either as homozygotes (33.2%) or heterozygotes (16.2%) (Table 4). The frequency of the *kdr* allele was estimated at 51% in the population.

**Table 4:** Frequencies of *Kdr* (West Africa) mutation in *Cx quinquefasciatus* population from Yaoundé.

Insecticides	*Kdr* mutation			
	SS	RS	RR	F(R)
0.05% Deltamethrin (n = 116)	39	19	58	0.58
0.75% Permethrin (n = 78)	39	19	20	0.38
4% DDT (n = 7)	1	2	4	0.59
Total	79	40	82	0.51

#### Infections by *Wuchereria bancrofti*

Out of 247 mosquito pools, each comprising 25 Culex mosquitoes, none were found to be infected with Wuchereria bancrofti. No positive control was available, however we applied a published protocol. We tested 4 pools of 25 newly emerged mosquitoes as a negative control and all tested negative.

### Discussion

The study objective was to assess culicine species distribution, bionomic and potential role in *W*. *bancrofti* transmission in the city of Yaoundé. High Culicine species diversity was recorded with up to 13 species collected. *Culex* species were the most prevalent and this was consistent with previous studies conducted in Cameroon and across Africa indicating the high adaptation capacity of species of this genus particularly *Cx*. *quinquefasciatus* to the urban environment [27, 41–44]. The diversity of culicine species recorded could result from the presence of different landscapes across the city of Yaoundé made up of an alternation of highland and marshland covered with vegetation and exploited for agriculture, lakes invaded by vegetation, and rural environment. It is still unknown whether there is an intense competition between culicine species sharing similar habitats. Species such as *Cx*. *tigripes* larvae are known to be predators for early instars of different species. *Culex quinquefasciatus* emerged after molecular analysis, as the sole member of the *Cx*. pipiens complex in Yaoundé; its presence was consistent with the known distribution of members of the complex [37].

Species diversity and abundance were all found to vary according to collection methods and seasons. High species diversity was recorded using CDC-LT compared to HLC or larval collection and reflects the high efficiency of CDC-LT method for collecting culicines. The use of CDC-LT has now become common for sampling mosquito populations across the world and has been shown to be particularly effective for sampling *Culex* mosquitoes [27, 45]. This tool was rather found to underestimate anophelines densities [27, 45, 46]. Both HLC and CDC-LT techniques were used because there was so far no available data on the efficiency of CDC-LT for collecting Culex species from Yaoundé.

Seasonal variations in species composition was detected for mosquitoes collected from breeding habitats, however, no similar trend was detected for mosquitoes collected using CDC-LT or HLC. This likely suggest different breeding habitats preference for culicine species at different periods of the year or the influence of physico-chemical parameters [47, 48] or xenobiotics selection [49] on *Culex* species distribution. *Cx*. *quinquefasciatus* larvae were found to be highly prevalent in polluted sites. It is likely that females of *Culex* species are more attracted by oviposition cues released by the microbial fauna in this type of habitats. In addition, these habitats are rich in nutrients and could thus reduce competition for resources between species. This could also be because mosquitoes in polluted sites are also frequently exposed to intensive selective pressure induced by pollutants and xenobiotics [27, 50–52], different strategies were reported to promote *Culex* species adaptation to different ecological constraints. This include the development of resistance or detoxification mechanisms to a large set of insecticides and xenobiotics [53–55], the capacity for eggs to resist desiccation [56] and development of cuticle resistance in larvae [3, 57, 58]. Several *Culex* species including *Cx*. *quinquefasciatus*, *Cx*. *antennatus*, *Cx*. *duttoni* were found to display resistance to DDT, permethrin and deltamethrin. This is the first time that insecticide resistance in different *Culex* species is documented in Cameroon. The level of pyrethroid resistance was similar to data recorded for *An*. *gambiae* populations in the city of Yaoundé [59, 60]. In addition to the fact that *Culex* species are known to breed in polluted environment and could thus be affected by xenobiotics selection, the high level of resistance recorded could also result from increased use of LLINs for malaria vector control and pesticides use in agriculture in the city of Yaoundé [27, 61]. Our study also suggested the presence of *kdr* allele in *Cx*. *quinquefasciatus* populations. It is likely that resistance in *Culex* species is sustained by both *kdr* mutations and other mechanisms such as the metabolic detoxification machinery [62].

The present study also permitted to evaluate the role of *Culex* species in LF transmission after mass drug administration (MDA) scale up in Cameroon. *Culex quinquefasciatus* is the predominant vector of LF in both urban and rural settings in East Africa [3, 23] but less so in Central and West Africa. However, with potential gene flow and changing climate, one cannot rule out that *Cx*. *quinquefasciatius* in Central Africa such as in Cameroon may also emerge as LF vector. Furthermore, because of the rapid expansion and predominance of this species in Cameroon cities, it’s potential implication in LF transmission in Yaoundé was examined. Analysis conducted with pool samples of *Culex* mosquitoes recorded no infection. In Cameroon LF is considered to be endemic with prevalence rates varying from 1 to 8% [19, 63]. It is likely that the prevalence of parasite may have decreased over years due to the implementation of mass drug administration of ivermectin and abendazole to the population since 2009 [19]. So far, five to six rounds of MDA have been successfully conducted in endemic settings across the country and interruptions of LF transmission have been documented in some parts of the country [64]. The fact that only Culex species were screened during this study could have limited the capacity of detecting any ongoing transmission since mosquito species such as *An*. *gambiae* and *An*. *funestus* are also good vectors of LF [3, 23]. Another important dimension which could explain the absence of *W*. *bancrofti* infection in *Culex* is that the area may have not been endemic for *W*. *bancrofti* before the introduction of MDA. Recent studies conducted in Cameroon and DRC suggested that the perceived endemicity of LF established by ICT test in the central African region could result from the presence of *Loa* filariasis which cross react to the ICT tests which was used to detect *W*. *bancrofti* in Central Africa, leading to false positivity [64–66]. During the last decade, several arboviral diseases such as chikungunya, dengue, yellow fever, West Nile, Sindbis, Tahyna, O’nyong-nyong and spondweni virus have been reported in circulation in human adults in both urban and rural settings [67–70]. With the rapid distribution of *Culex* species in the urban environment, the potential role that these species could play in spreading of these arboviral diseases deserves further consideration.

### Conclusion

The present study confirms high abundance of *Cx*. *quinquefasciatus* in the city of Yaoundé and high insecticide resistance in most *Culex* species populations. The study also suggests no transmission of *W*. *bancrofti* by *Culex* species in Yaoundé. In Cameroon, apart from malaria vectors, surveillance activities are not regularly conducted on other vectors of diseases because of lack of funding or technical capacities for these activities. In this context, combining surveillance activities of malaria vectors with other culicine species and strengthening capacities of medical entomologists on taxonomy, sampling, processing and calculation of key entomological indicators for endemic vector borne diseases could be cost effective and will enable better understanding of the distribution and epidemiology of various diseases. This could lead to the establishment of sustainable surveillance systems.

## Supporting information

S1 Data Distribution of culicine species in different districts of the city of Yaoundé. (DOCX)
