# Supplementary material for: Culex species diversity, susceptibility to insecticides and role as potential vector of Lymphatic filariasis in the city of Yaoundé, Cameroon
Source: PLoS Negl Trop Dis. 2019 Apr 3;13(4):e0007229. doi: 10.1371/journal.pntd.0007229 (PMC6464241; doi:10.1371/journal.pntd.0007229)
Supplement: S1 Data — (DOCX) [file pntd.0007229.s001.docx]

Supplement data 1 : Distribution of culicine species in different districts of the city of Yaoundé

|  | *Culex* | | | | | | | *Aedes* | | | *Mansonia* | | *Coquilletidia* | Total |
| --- | --- | --- | --- | --- | --- | --- | --- | --- | --- | --- | --- | --- | --- | --- |
|  | *Cx. quinquefasciatus* | *Cx. perfuscus* | *Cx. duttoni* | *Cx. tigripes* | *Cx. poicilipes* | *Cx. antennatus* | *Cx. univittatus* | *Ae. albopictus* | *Ae. aegypti* | *Ae. furcifer* | *Man. uniformis* | *Man. africanus* | *Coquelletidia sp* |  |
| 1. Ambassade France | 250 | 40 | 15 | 3 | 0 | 0 | 0 | 0 | 3 | 0 | 0 | 12 | 5 | 328 |
| 2. Biyem-assi Carrefour | 250 | 33 | 19 | 1 | 0 | 0 | 0 | 0 | 1 | 1 | 0 | 0 | 0 | 305 |
| 3. Biyem-assi Lac | 317 | 36 | 0 | 0 | 0 | 4 | 0 | 0 | 2 | 0 | 0 | 0 | 0 | 359 |
| 4. Biyem-assi Lycée | 340 | 13 | 18 | 3 | 0 | 0 | 0 | 0 | 11 | 4 | 0 | 1 | 0 | 390 |
| 5. Biyem-assi SOMATEL | 250 | 5 | 6 | 0 | 0 | 0 | 0 | 0 | 2 | 30 | 0 | 6 | 2 | 301 |
| 6. Cité des Nations | 250 | 26 | 3 | 0 | 0 | 0 | 0 | 0 | 0 | 0 | 0 | 1 | 0 | 280 |
| 7. Efoulan lac | 223 | 21 | 7 | 2 | 0 | 14 | 0 | 0 | 2 | 1 | 3 | 0 | 0 | 273 |
| 8. Etam Bafia | 507 | 29 | 43 | 0 | 0 | 21 | 0 | 0 | 2 | 0 | 0 | 42 | 4 | 648 |
| 9. Etoug-Ebe | 348 | 46 | 186 | 2 | 0 | 13 | 0 | 0 | 2 |  | 0 | 7 | 0 | 604 |
| 10. Essos | 417 | 77 | 31 | 0 | 0 | 0 | 0 | 0 | 6 | 10 | 0 | 0 | 0 | 541 |
| 11. GP melen | 336 | 3 | 22 | 4 | 0 | 0 | 0 | 0 | 0 | 2 | 0 | 23 | 0 | 390 |
| 12. Labogénie | 250 | 15 | 10 | 0 | 0 | 2 | 0 | 0 | 0 | 0 | 3 | 39 | 0 | 319 |
| 13. Mvog-Ada | 266 | 10 | 14 | 0 | 0 | 19 | 0 | 0 | 1 | 0 | 0 | 5 | 0 | 315 |
| 14. Nkolbikok | 320 | 25 | 23 | 1 | 0 | 2 | 0 | 0 | 3 | 0 | 0 | 8 | 0 | 382 |
| 15. Nkoldongo | 291 | 20 | 6 | 1 | 0 | 2 | 4 | 4 | 0 | 0 | 0 | 4 | 0 | 332 |
| 16. NR Bastos | 278 | 10 | 9 | 0 | 0 | 3 | 0 | 0 | 10 | 4 | 0 | 0 | 0 | 314 |
| 17. NR Tam-tam | 723 | 49 | 47 | 3 | 1 | 0 | 0 | 0 | 1 | 0 | 0 | 81 | 3 | 908 |
| 18. Nsam | 413 | 94 | 19 | 3 | 0 | 18 | 0 | 0 | 3 | 8 | 0 | 12 | 0 | 570 |
| 19. Olézoa | 286 | 13 | 26 | 0 | 0 | 2 | 0 | 0 | 3 | 0 | 0 | 0 | 0 | 330 |
| 20. Santa Barbara | 327 | 19 | 37 | 2 | 0 | 1 | 0 | 0 | 4 | 0 | 0 | 0 | 0 | 390 |
| 21.Shell Obili | 334 | 7 | 3 | 0 | 0 | 0 | 0 | 0 | 10 | 0 | 0 | 1 | 2 | 357 |
| 22. Emia | 416 | 18 | 15 | 6 | 0 | 0 | 0 | 0 | 7 | 0 | 0 | 4 | 5 | 471 |
| 23. Tsinga | 261 | 10 | 18 | 0 | 0 | 0 | 0 | 0 | 5 | 3 | 0 | 8 | 0 | 305 |
| 24/ Ekounou Palais | 379 | 77 | 46 | 4 | 1 | 253 | 0 | 0 | 2 | 0 | 0 | 83 | 17 | 862 |
| 24. Ekounou Ekié | 283 | 45 | 11 | 1 | 0 | 80 | 0 | 0 | 2 | 1 | 0 | 36 | 4 | 463 |
| 26. Ngousso | 264 | 48 | 9 | 6 | 1 | 3 | 0 | 0 | 5 | 0 | 0 | 83 | 17 | 436 |
| 27. NR Nkolbisson | 245 | 48 | 74 | 0 | 0 | 0 | 0 | 0 | 6 | 0 | 0 | 4 | 0 | 377 |
| 28. Obobogo | 283 | 26 | 4 | 0 | 0 | 0 | 0 | 0 | 0 | 0 | 0 | 4 | 0 | 317 |
| 29. Oyom-Abang | 250 | 37 | 28 | 1 | 0 | 0 | 0 | 0 | 12 | 0 | 0 | 5 | 1 | 334 |
| 30. Nkolbisson | 334 | 20 | 296 | 40 | 0 | 21 | 0 | 0 | 3 | 0 | 0 | 16 | 0 | 730 |
| 31. Mendong | 414 | 65 | 58 | 0 | 0 | 9 | 0 | 0 | 5 | 1 | 0 | 51 | 5 | 608 |
| 32. Tongolo | 250 | 44 | 3 | 0 | 0 | 144 | 0 | 0 | 2 | 0 | 0 | 0 | 0 | 443 |
| Total | 10355 | 1029 | 1106 | 83 | 3 | 611 | 4 | 4 | 115 | 65 | 6 | 536 | 65 | 13982 |
| % | 74.05 | 7.35 | 7.91 | 0.6 | 0.02 | 4.37 | 0.03 | 0.03 | 0.82 | 0.46 | 0.04 | 3.83 | 0.46 | 100 |
